# Functional Rescue of a Nephrogenic Diabetes Insipidus Causing Mutation in the V2 Vasopressin Receptor by Specific Antagonist and Agonist Pharmacochaperones

**DOI:** 10.3389/fphar.2022.811836

**Published:** 2022-01-25

**Authors:** Laura Szalai, András Sziráki, László Sándor Erdélyi, Kinga Bernadett Kovács, Miklós Tóth, András Dávid Tóth, Gábor Turu, Dominique Bonnet, Bernard Mouillac, László Hunyady, András Balla

**Affiliations:** ^1^ Department of Physiology, Semmelweis University, Budapest, Hungary; ^2^ MTA-SE Laboratory of Molecular Physiology, Eötvös Loránd Research Network and Semmelweis University, Budapest, Hungary; ^3^ Department of Internal Medicine and Oncology, Semmelweis University, Budapest, Hungary; ^4^ Department of Internal Medicine and Haematology, Semmelweis University, Budapest, Hungary; ^5^ Laboratoire D'Innovation Thérapeutique, Strasbourg Drug Discovery and Development Institute (IMS), UMR7200 CNRS, Université de Strasbourg, Illkirch-Graffenstaden, France; ^6^ Institut de Génomique Fonctionnelle, Université de Montpellier, CNRS, INSERM, Montpellier, France; ^7^ Institute of Enzymology, Research Centre for Natural Sciences, Budapest, Hungary

**Keywords:** vasopressin receptor 2 (V2R), nephrogenic diabetes insipidus (NDI), bioluminescence resonance energy transfer (BRET), pharmacochaperone, tolvaptan, G protein coupled receptor (GPCR), arginin vasopressin, MCF-14

## Abstract

The urine concentrating function of the kidney is essential to maintain the water homeostasis of the human body. It is mainly regulated by the arginine-vasopressin (AVP), which targets the type 2 vasopressin receptor (V2R) in the kidney. The inability of V2R to respond to AVP stimulation leads to decreased urine concentration and congenital nephrogenic diabetes insipidus (NDI). NDI is characterized by polyuria, polydipsia, and hyposthenuria. In this study, we identified a point mutation (S127F) in the *AVPR2* gene of an NDI patient, and we characterized the impaired function of the V2R mutant in HEK293 cells. Based on our data, the S127F-V2R mutant is almost exclusively located intracellularly in the endoplasmic reticulum (ER), and very few receptors were detected at the cell surface, where the receptor can bind to AVP. The overexpressed S127F-V2R mutant receptor has negligible cAMP generation capability compared to the wild-type receptor in response to AVP stimulation. Since certain misfolded mutant proteins, that are retained in the ER, can be rescued by pharmacological chaperones, we examined the potential rescue effects of two pharmacochaperones on the S127F-V2R. We found that pretreatment with both tolvaptan (an established V2R inverse agonist) and MCF14 compound (a cell-permeable high-affinity agonist for the V2R) were capable of partially restoring the cAMP generating function of the receptor in response to vasopressin stimulation. According to our data, both cell permeant agonists and antagonists can function as pharmacochaperones, and serve as the starting compounds to develop medicines for patients carrying the S127F mutation.

## Introduction

The antidiuretic hormone arginine-vasopressin (AVP) and its type 2 vasopressin receptor (V2R) in the kidney, are the main effectors in the regulation of the urine concentration and hence the maintenance of body water homeostasis (Robertson, 2001). AVP is released from the posterior pituitary in response to either decreased blood volume and/or arterial blood pressure or increased plasma osmolality (Juul et al., 2014). The main effect of the circulating AVP is binding to V2R on the basolateral membrane of the collecting duct principal cells. The AVP binding initiates the redistribution of aquaporin 2 (AQP2) water channels from intracellular vesicles to the apical membrane (Moeller et al., 2013). By this mechanism, AVP increases water permeability in the collecting duct, essential for urinary concentration (Nielsen et al., 1993). The V2R is a member of the G protein-coupled receptor (GPCR) superfamily. AVP binding activates the G_s_ heterotrimeric G protein, which stimulates adenylyl cyclase, thus generating a second messenger, 3′,5′-cyclic adenosine monophosphate (cAMP). The cAMP signal induces the fusion of intracellular vesicles containing APQ2 proteins with the apical surface of the duct cells (Boone and Deen, 2008). On the contrary, declining AVP concentration in the blood plasma retrieves the AQP2 water channels back into the cytosol via endocytosis, and the water reabsorption returns to a lower rate. AVP promotes urine concentration not only by increasing the water permeability of the collecting ducts but also by enhancing the urea permeability of the inner medullary collecting ducts promoting a corticopapillary interstitial osmotic gradient favoring the water reabsorption. Various mutations in the arginine vasopressin type 2 receptor gene (*AVPR2*) can result in loss-of-functionV2Rs, causing either impaired receptor signal transduction or decreased number of cell surface receptors. These impaired receptors lead to congenital X-linked nephrogenic diabetes insipidus (NDI), which causes several clinical manifestations such as polyuria, polydipsia, and hyposthenuria (Bichet et al., 1994; Erdelyi et al., 2014). On the contrary, gain-of-function mutations of the V2R can lead to the nephrogenic syndrome of inappropriate antidiuresis disease (NSIAD) (Feldman et al., 2005; Erdelyi et al., 2015). Up to present, more than 200 mutations, which most frequently occur in transmembrane domains of the receptor, have been described and various mechanisms are responsible for the impaired functions of the mutated receptors (Bichet and Bockenhauer, 2016; Balla and Hunyady, 2019).

The cellular manifestations of the V2R mutations can be classified into four main categories (Wesche et al., 2012): the class I mutations of the *AVPR2* gene prevent the effective synthesis of the V2R, the class II V2R mutants are structurally disrupted and retained in intracellular compartments, the class III mutant receptors reach the cell surface, but they display either reduced G protein coupling in response to AVP stimulation or impaired ligand affinity, whereas the class IV mutants show decreased basolateral surface expression because of the V2R accumulation in intracellular vesicles due to their altered intracellular trafficking. The class II V2R mutations are the most frequently occurring ones, caused by missense/nonsense mutations that lead to the formation of misfolded full-length or truncated proteins. These mutant receptors are recognized by the quality control system of the endoplasmic reticulum (ER), resulting in defective intracellular trafficking of the V2R mutants. They are usually retained in ER, and the degradation of the trapped receptors often occurs (Robben et al., 2006).

In some cases, misfolded receptors can be rescued by cell permeant pharmacological chaperones, which can bind to the receptor in the ER, and can stabilize the conformation sufficiently to pass the quality control system (Ulloa-Aguirre et al., 2004). Although these pharmacochaperones can induce targeting of the receptors at the cell surface, most of them are antagonists, and their continuous presence can block the function of the rescued receptors. This problem may be reduced by the administration of cell permeant V2R agonists, if they can rescue the function of the mutant receptor. Earlier studies have indicated that in case of some mutation, rescue of the pathogenic mutant V2 receptors can be performed with non-peptide agonists. In the present study, we have investigated whether plasma membrane targeting of the S127F-V2Rs can be performed using a V2R agonist and a V2R antagonist.

## Materials and Methods

### Materials

Molecular biology enzymes were brought from Fermentas (Burlington, Canada), Stratagene (La Jolla, CA, United States), and Invitrogen (Carlsbad, CA, United States). Coelenterazine *h* was purchased from Regis Technologies (Morton Grove, IL). Cell culture dishes and plates for BRET measurements were purchased from Greiner (Kremsmunster, Austria), whereas the plates for microscopy measurements were obtained from IBIDI (Martinsried, Germany). Lipofectamine 2000 and anti-HA-Alexa488 monoclonal antibodies were purchased from Invitrogen (Carlsbad, CA, United States). Unless otherwise stated, all other chemicals and reagents were purchased from Sigma-Aldrich (St. Louis, MO, United States).

### Mutation Analysis of the AVPR2 Gene

Family history, clinical and laboratory data, and signed informed consent were obtained from the parents of the index patient. The sequencing method of the *AVPR2* gene was the same as described previously (Erdelyi et al., 2014). Briefly, the *AVPR2* gene was amplified from genomic DNA samples obtained from peripheral blood leukocytes using a DNA isolation kit (Boehringer Mannheim Corporation, Indianapolis, IN, United States) and the entire coding region of the gene was Sanger sequenced in both directions (Eurofins MWG Operon, Ebersberg, Germany). The database sequence NM_000,054 was used as the reference sequence for the *AVPR2* gene.

### Molecular Biology

The plasmid constructs (untagged and HA-tagged) coding the wild-type V2R were described previously (Erdelyi et al., 2014). For the generation of the S127F-V2R plasmid constructs standard site-directed mutagenesis techniques were performed. To avoid incidental mutations, after verifying the mutations with dideoxy sequencing, the mutated fragment was exchanged between the wild-type and mutated portion with suitable restriction sites to avoid the generation of unwanted mutations outside the sequenced regions.

### Cell Culture and Transfection

The human embryonic kidney (HEK293) cell lines were obtained from ATCC (American Type Culture Collection, Manassas, VA, United States). The cells were cultured in DMEM (Biosera, Nuaille, France) supplemented with 10% heat-inactivated fetal bovine serum and 100 IU/ml penicillin/streptomycin (Invitrogen) in 5% CO_2_ at 37°C. The cells were cultured in plastic dishes and were trypsinized before transfection. The cells were transiently transfected by using Lipofectamine 2000 (Invitrogen) according to the manufacturer’s instructions, and after 6 hours, the transfection medium was replaced with complete DMEM. For bioluminescence resonance energy transfer (BRET) measurements, the HEK293 cells were transfected in suspension, and the cells were plated on white 96-well plates. The amount of Lipofectamine 2000 was 0.5 μL/well, whereas the DNA amounts were 0.25 μg receptor-containing construct and 0.25 μg Epac-BRET biosensor/well. For measurement of receptor expression in flow cytometry, the HEK293 cells were transiently transfected with either HA-tagged V2-receptor construct or pcDNA3.1 for 24 h (6 μg of DNA and 12 μL Lipofectamine 2000/100 mm dish). For microscopy determinations, the cells were plated onto poly-lysine (Sigma) pretreated 24-well plates at a density of 5 × 10^4^ cells per well or µ-Slide 8-well plates at a density of 3.5 × 10^4^ cells per well in the day before the transfection. The adherent cells were transfected with the appropriate vectors (pCDNA3.1, WT-V2R-HA, or S127F-V2R-HA vectors) using 0.5 μg (in case of 24-well plates) or 0.15 μg (in case of 8-well plates) of DNA and Lipofectamine 2000 transfection reagent.

### Bioluminescence Resonance Energy Transfer Measurements

The BRET measurements were performed on adherent cells after 24 h of the transfection on white 96-well plates. Before the measurements, the cells were washed and the medium was changed to a modified Kreb’s-Ringer buffer containing 120 mM NaCl, 4.7 mM KCl, 1.8 mM CaCl_2_, 0.7 mM MgSO_4_, 10 mM glucose, and Na-HEPES 10 mM, pH 7.4; and all measurements were performed at 37°C. In the case of pharmacochaperone pretreatment studies, before the BRET measurements of the pretreated cells, the medium of the cells was replaced every 15 min for 1 hour to wash out the remnants of tolvaptan or MCF14. The Epac-BRET intramolecular probe was constructed in our earlier study (Erdelyi et al., 2014). The BRET measurements were started after the addition of the cell-permeable substrate, coelenterazine *h* at a final concentration of 5 μM. The counts were recorded by using a Varioskan Flash multimode plate reader (Thermo Scientific, Carlsbad, CA, United States) using filters at 480 and 530 nm wavelengths. The detection time was 0.25–0.5 s. The baselines were detected for ten cycles, and the cells were stimulated with either vehicle or receptor agonist. The BRET ratios were calculated as 530 nm/485 nm ratio. Measurements were done in triplicate. The BRET records are average of at least three independent experiments. Curve fittings were performed with GraphPad Prism software.

### Immunofluorescence Staining

After 24 h of the transfection, the cells were treated with vehicle (DMSO), tolvaptan, or MCF14 for 18 h. Subsequently, immunofluorescence staining was performed on non-permeabilized or permeabilized cells while keeping the plates on ice. For immunostaining of non-permeabilized cells, the HEK293 cells were carefully washed once, incubated with anti- HA-Alexa488 conjugated antibody in 1:500 dilution for 1 h, fixed with 3.7% paraformaldehyde solution for 15 min, and washed three times for 10 min with modified Kreb’s-Ringer solution. The immunostaining of the permeabilized cells was performed according to the following protocol: the transfected cells were gently washed once before fixation with 3.7% paraformaldehyde solution for 15 min. After quenching the fixation solution by three washing steps, the cells were permeabilized using 0.1% Triton X-100 (Sigma) and incubated in 0.1% sodium-borohydride solution for 15 min. The cells were then incubated in 1% BSA (Sigma) containing blocking solution for 30 min, and immunolabelled using anti-HA-tag Alexa488 conjugated antibody in 1:500 dilution for 1 h. Lastly, cells were washed three times for 10 min with modified Kreb’s-Ringer solution.

### Microscopy

Imaging of 24-well plates was executed with ImageXpress Micro Confocal microscope (Molecular Devices, San Jose, CA, United States), keeping the same exposure parameters constant (acquisition time and gain) between wells. The microscopic images were obtained by using MetaXpress software. The cell fluorescence was determined by the corrected total cell fluorescence (CTCF) method using ImageJ software (Schindelin et al., 2012). The localization and distribution of the HA-tagged receptors in 6-well or Ibidi plates were analyzed using a Zeiss LSM 710 (Oberkochen, Germany) confocal laser-scanning microscope.

### Flow Cytometry

The cell-surface expression levels of the receptors were analyzed and quantified by flow cytometry using BD FACSCalibur (Franklin Lakes, NJ, United States). The transfected cells were treated with vehicle or tolvaptan or MCF14 for 18 h. Before the measurement, the cells were detached by aspirating the medium and treatment with Versene solution. The detached cells were collected by gentle centrifugation and suspended at a density of 1 × 10^6^ cells/ml in ice-cold PBS and centrifuged at 4°C. The cell pellets were suspended and incubated with diluted (1:50) anti-HA-Alexa488 mouse monoclonal antibody for 40 min on ice. The unbound antibodies were removed by three rounds of washing with ice-cold PBS and centrifugation at 4°C. Non-specific fluorescence was determined by measuring empty pcDNA3.1 transfected HEK293 cells incubated with anti-HA-Alexa 488 antibodies. Fluorescence data were collected using logarithmic amplification until 10^3^ counts were reached, median fluorescence values were analyzed and the fluorescent intensity values were plotted using the GraphPad Prism software.

### Statistical Analysis

The statistical analysis was performed with GraphPad Prism software. For statistical comparison, one-way ANOVA with Dunnett posthoc test was applied. Mean values ±S.E. of at least three independent experiments are shown in the figures.

## Results

### Identification of the S127F Mutation in the AVPR2 Gene

**FIGURE 1:**
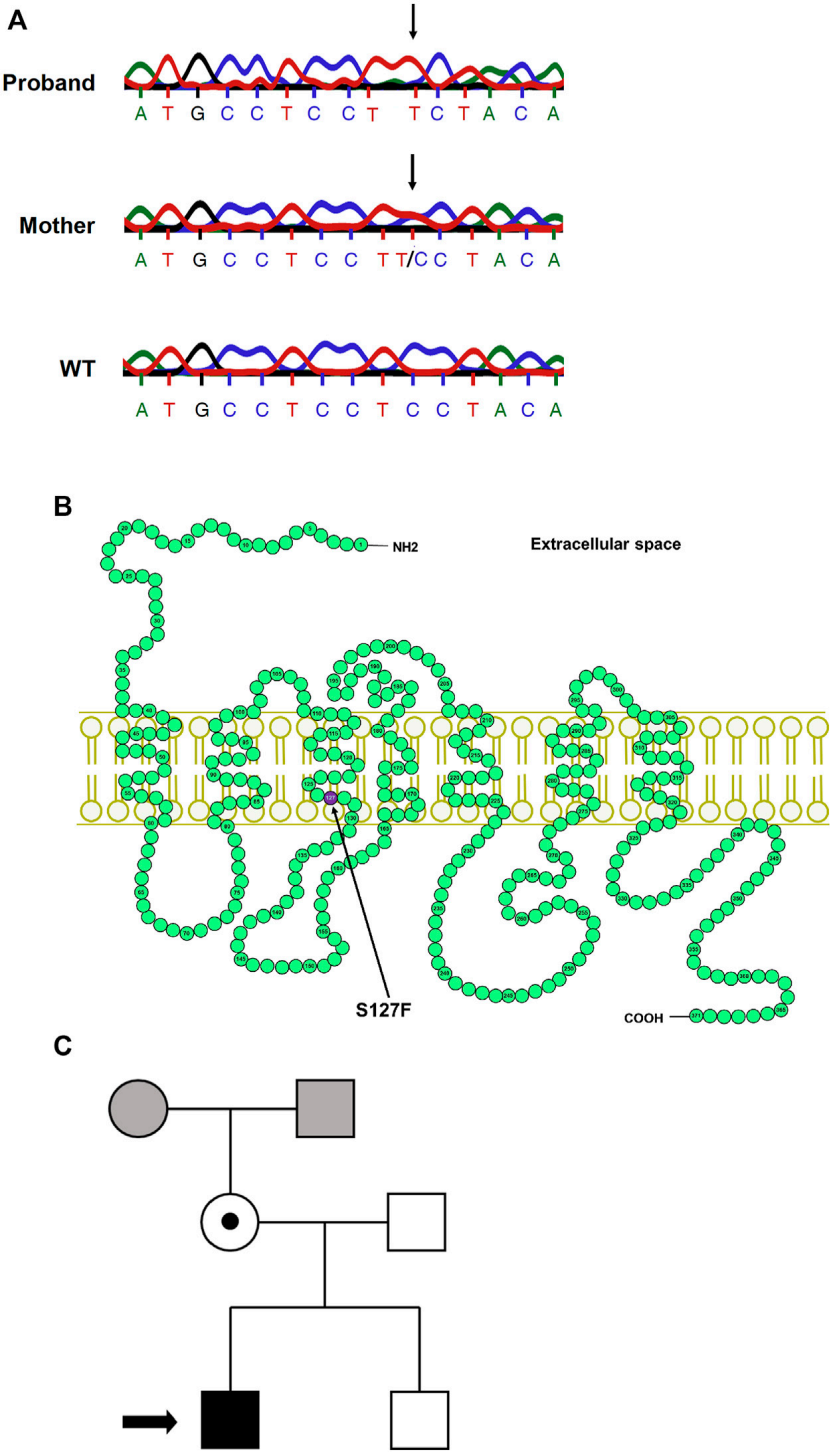
Mutation analysis of the *AVPR2* gene and the family pedigree. **(A)** The chromatogram of the Sanger sequencing of *AVPR2* gene from the proband (upper panel), the mother of the proband (middle panel), and a healthy control individual (lower panel). The arrows indicate the mutation in the DNA sequences. **(B)** The schematic representation of the human V2 vasopressin receptor. The arrow indicates the affected amino acid (purple circle) in the third transmembrane helix of the V2R. **(C)** The arrow in the pedigree of the family indicates the proband, the solid black square represents the male individual with the classical symptoms of NDI whereas the heterozygous mother with subclinical NDI is represented by an open circle with a central dot. The mutation was not found in the brother of the proband, the genotypes of the maternal parents are not known (grey square and circle).

The proband male patient was born in 2012 and was diagnosed with NDI during infancy. He manifested the classical signs and symptoms of NDI (polyuria and polydipsia; the daily urine production was around 1 L of the ∼5,000 g infant). The clinical diagnosis of the NDI was confirmed by a water deprivation test and by the administration of 1-deamino-8-d-arginine-vasopressin (dDAVP, desmopressin), which was ineffective. The mother was born in 1981 and had no severe symptoms; her subclinical NDI was not diagnosed until the investigation of her infant. Her average estimated fluid intake is 3–4 L/day, and she is in good general health. We performed a molecular mutation analysis of the *AVPR2* gene after PCR amplification of DNA samples isolated from the peripheral blood of the proband and his mother. The direct DNA sequencing of the PCR products revealed a point mutation in the proband (Figure 1A). This missense mutation caused a cytosine to thymine single-base substitution (g.741C→T), which resulted in an amino acid substitution from serine-127 to phenylalanine (S127F). No other mutation was found in the *AVPR2* gene. The sequencing revealed that this mutation is not a *de novo* mutation since the same mutation was found in the mother, as well. According to the DNA sequencing, the mother is a heterozygous carrier since both the wild-type (WT) and the mutant alleles were present in the DNA sample of the mother (Figure 1A, middle panel). The coding sequence of *AVPR2* is distributed on three exons and the identified g.741C→T point mutation is located in exon 2. The resulting S127F amino acid substitution is located in the third transmembrane domain of the V2 vasopressin receptor (Figure 1B). The symptoms of the NDI were not present in the patient’s younger brother (Figure 1C). The genotypes of the maternal grandparents are not known.

### Characterization of the Mutant Receptor

**FIGURE 2:**
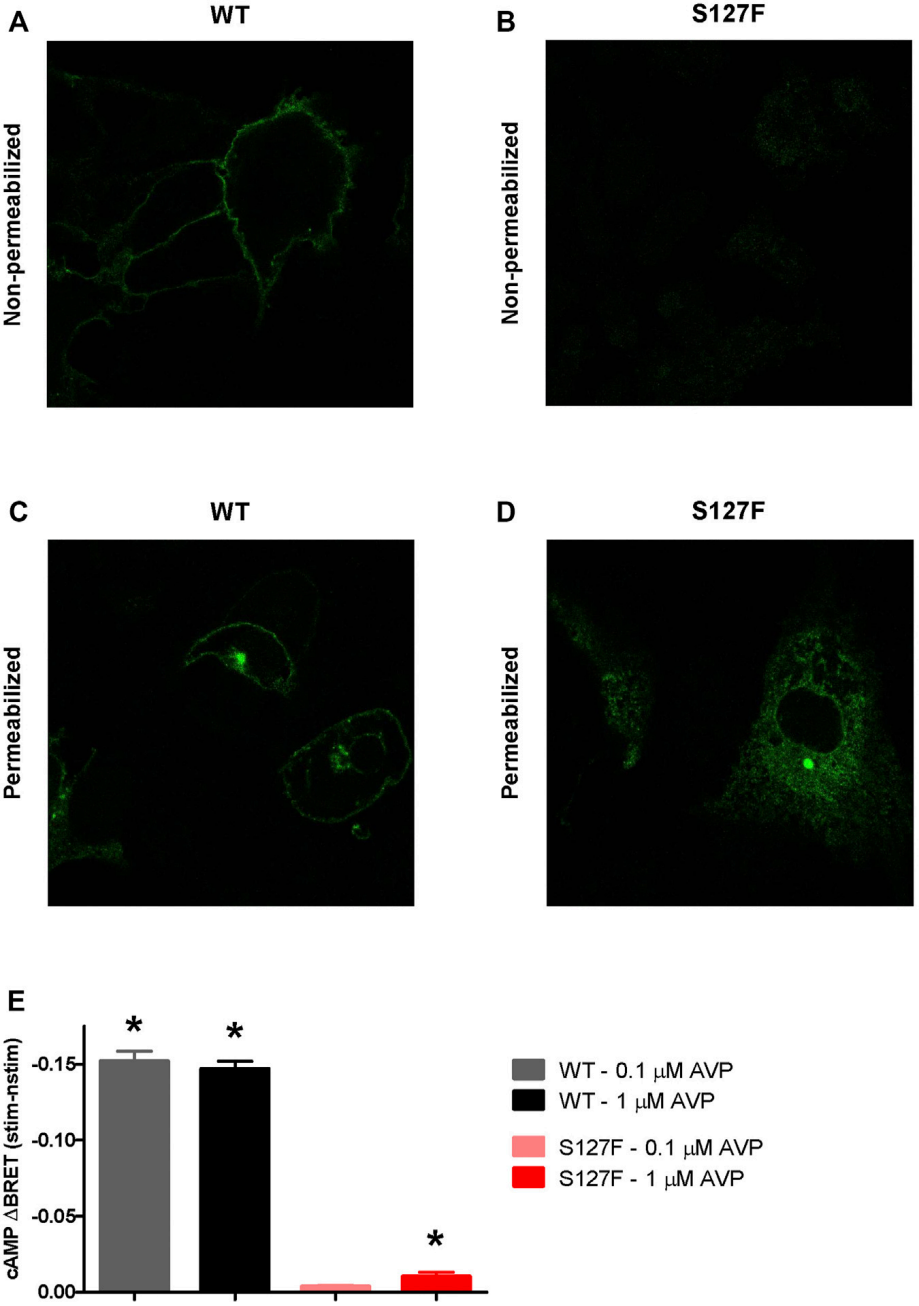
Characterization of the cellular localization and cAMP signaling of the wild-type and S127F mutant V2 receptors. **(A–D)** Examination of the cell surface and intracellular expression of the wild-type and S127F V2Rs using confocal microscopy. HEK293 cells were transiently transfected with the plasmids of WT-V2R-HA or S127F-V2R-HA. The HA-tag-containing receptor variants were directly immunostained with anti-HA-Alexa488 mouse monoclonal antibody. **(A)** WT-V2R and **(B)** S127F-V2R presence at the cell surface was analyzed after immunostaining of non-permeabilized fixed cells, whereas the total cellular distribution of **(C)** WT-V2R and **(D)** S127F-V2R was assessed after staining permeabilized cells. **(E)** Measurement of cAMP production after AVP stimulation. HEK293 cells were transiently transfected with the plasmids of the Epac-BRET sensor and either WT-V2R or S127F-V2R. The cells were treated either with vehicle (nstim) or with the indicated concentration of AVP (stim) and the BRET ratios were monitored. The effect of stimulation was calculated as the BRET ratio difference between the AVP (stim) and the vehicle (nstim) exposed cells after 500 s of treatment. Mean values ±S.E. are shown (*n* = 3). Significance was calculated with one-way ANOVA test (*p < 0.05).

The S127F mutation was already identified in a Japanese family as putative disease-causing AVPR2 mutation, but no characterization of the functional consequences of the mutation were performed (Arthus et al., 2000). We decided to characterize the impaired functions of this missense mutation (S127F). First, we wanted to establish the mechanism of how the mutation leads to disease. Second, we aimed to restore the function of the mutant receptor. The majority of missense *AVPR2* gene mutations result in class II, ER retention mutant receptors (Wesche et al., 2012; Balla and Hunyady, 2019), although it is important to establish the cellular consequences for every new mutation. Therefore, we transiently overexpressed HA-tagged S127F mutant and WT human V2 vasopressin receptors in HEK293 cells. The expressed receptors were immunolabeled by anti-HA-Alexa488 monoclonal antibodies, and the fluorescence was detected with confocal microscopy. We found that the WT-V2R was located in the plasma membrane (Figure 2A), whereas the HA-tagged S127F mutant V2R was not detectable on the cell surface (Figure 2B), proving that the mutation is not in the class III group. Next, we investigated whether the lack of the mutant receptor on the cell surface is the consequence of one the two following hypotheses. The ineffective synthesis of the V2R (class I: impaired transcription, mRNA processing, or translation of the receptor) or misfolding of the full-length receptor formation (class II), which is recognized by the quality control system of the ER that triggers intracellular retention and leads to the absence of the receptor on the cell surface. To determine the type of the mutation, we permeabilized the HA-tagged receptor overexpressing cells to label the intracellular receptors with immunofluorescent anti-HA antibodies. The WT-V2R showed some intracellular staining, but was mainly present in the plasma membrane (Figure 2C). The small intracellular fraction of the transiently expressed WT receptors are in the secretory transport route to mature and reach the plasma membrane (Figure 2C). The marked intracellular staining of the S127F-V2Rs revealed that the mutant receptor is synthesized, but not able to reach the cell membrane due to intracellular trapping (Figure 2D). Finally, we were able to rule out the possibility of a class IV mutation, because the intracellular localization pattern of the mutant receptors is characteristic of the ER but not the intracellular vesicles (Figure 2D). Taken together, the S127F-V2R was synthesized, almost exclusively located in the intracellularly but not in a remarkable amount on the cell surface, indicating that the S127F-V2R is a class II, ER retention mutant.

Next, we were interested if the mutant receptors can be activated. Although the receptor surface expression is extremely low, in some cases amplification in the G_s_ protein-coupled receptor signaling cascades potentially enables the measurement of the downstream mediator cAMP, if the receptor can be activated. We compared the cAMP production of the wild-type and S127F mutant V2Rs in response to AVP stimulations using living cell bioluminescence resonance energy transfer (BRET) based experiments. A sensitive Epac-BRET intramolecular probe was used to monitor the intracellular cAMP level. cAMP binding initiates a conformational change in the sensor, shifting the relative positions of the energy acceptor and donor within the intramolecular probe, and changing the BRET ratio. The monitored BRET ratio changes can be followed in real-time by a BRET plate reader (Erdelyi et al., 2014). We transiently coexpressed the Epac-BRET probe with either WT-V2R or S127F-V2R, and the cAMP production was evaluated upon 0.1 and 1 µM AVP stimulation. The AVP stimulation of S127F-V2R expressing HEK293 cells caused only a minimal cAMP production compared to the cAMP signal generated by the WT receptor stimulation (Figure 2E). Although 1 µM AVP stimulation caused a very low degree of cAMP response, this minimal but statistically significant cAMP signal demonstrated partial functionality of the mutant receptor and raised the possibility to restore or improve the function of the S127F mutant V2 receptor.

### Functional Rescue of the S127F-V2R

**FIGURE 3:**
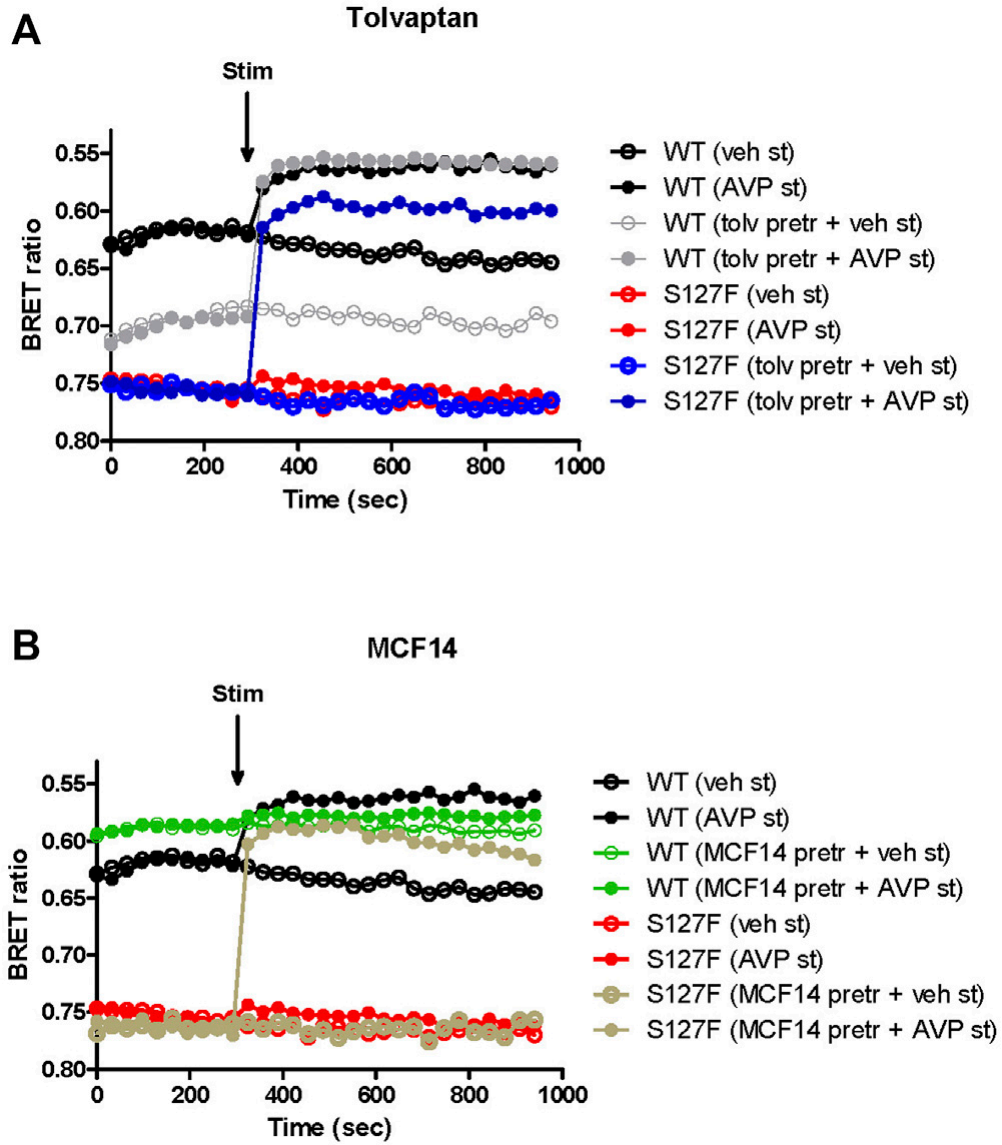
Effects of V2R ligand pretreatment on the cAMP generation of the WT-V2R or S127F-V2R. **(A)** Effect of tolvaptan or **(B)** MCF14 pretreatment on the intracellular cAMP production in response to the vehicle or AVP stimulation. HEK293 cells were transiently transfected with the plasmids of the Epac-BRET sensor and either WT-V2R or S127F-V2R. 24 h after the transfection 100 nM tolvaptan or 10 µM MCF14 or vehicle (DMSO) was added to the medium of the cells for 18 hours. Before the BRET measurements of the pharmacochaperone treated cells, the medium of the cells was replaced every 15 min for 1 hour to wash out the remnants of tolvaptan or MCF14. The cells were exposed to either vehicle (empty circles) or 1 µM AVP (filled circles) at the indicated time point. The BRET curves are an average of three independent experiments, each performed in triplicates. Mean values are shown but error bars were omitted for better clarity. Since increasing cAMP decreases the BRET ratio, the *y* axis was inverted to visually represent cAMP level changes.

Several V2R mutant variants were rescued by pharmacological chaperones, which can alter the conformation of the misfolded mutant receptors. Consequently, the intracellularly trapped receptors can be transported to the plasma membrane where the rescued receptors can bind AVP, the physiological ligand (Morello et al., 2000; Wuller et al., 2004; Bernier et al., 2006). First, we investigated the potential pharmacochaperone effect of tolvaptan. It was demonstrated previously that tolvaptan can rescue some of the misfolded and hence ER-retained V2R mutants (Robben et al., 2007; Takahashi et al., 2012; Makita et al., 2016). Tolvaptan is a good candidate as a pharmacochaperone because it is cell-permeable and specifically binds to V2R. WT-V2R or S127F-V2R expressing HEK293 cells were pretreated for 18 h. Strikingly different basal (before the agonist stimulation) BRET values were observed between the vehicle-treated WT-V2R (Figure 3, black traces) and S127F-V2R WT-V2R expressing cells (Figure 3, red traces). This reflects the constitutive activity of the V2Rs, and confirms the mutant receptor’s minimal expression on the cell surface. Despite the extensive washing steps starting 1 hour before the BRET measurements, the tolvaptan pretreatment reduced the basal activity of the wild-type receptor, showing its inverse agonist effect on cAMP generation (Figure 3A, grey traces, before the agonist stimulation). As expected, the tolvaptan did not affect the basal cAMP level in the cells expressing S127F-V2R, demonstrating that the mutant receptor expression did not cause any detectable constitutive activity (Figure 3A, blue and red traces, before the agonist stimulation). Upon 1 µM AVP stimulation the WT-V2R expressing cells showed a rapid and robust change in the BRET ratio, reflecting the increase in the intracellular cAMP level in both vehicle and tolvaptan pretreated cells (Figure 3A, black and grey filled circles). In contrast, the 1 µM AVP stimulation of the S127F-V2R expressing cells caused only minimal cAMP generation without tolvaptan pretreatment (Figure 3A, red filled circles) but the tolvaptan pretreatment significantly increased the cAMP response (Figure 3A, blue filled circles). This indicates the successful rescue of the mutant receptor by tolvaptan.

**FIGURE 4:**
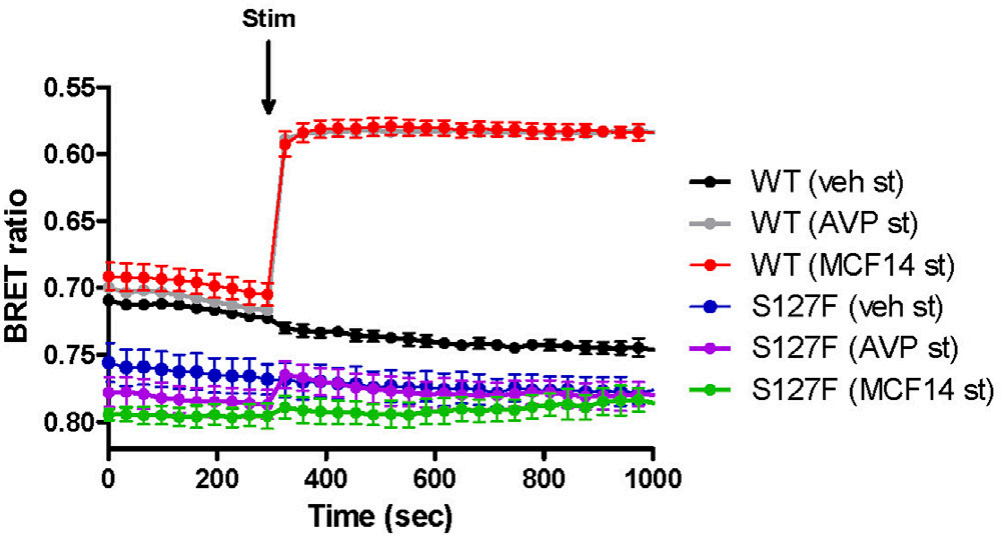
Effect of acute MCF14 stimulation on the cAMP level of wild-type or S127F mutant V2 receptor-expressing cells. HEK293 cells were transiently transfected for 24 h with the plasmids of the Epac-BRET sensor and either WT-V2R or S127F-V2R. The cells were exposed to vehicle or 1 µM AVP or 10 µM MCF14 at the indicated time point. The BRET curves are an average of three independent experiments, each performed in triplicates. Mean values ±S.E. are shown (*n* = 3). Since increasing cAMP decreases the BRET ratio, the *y* axis was inverted to visually represent cAMP level changes.

Second, we explored the functional rescue of the S127F-V2R using another type of V2R ligand, which was previously reported to act as a pharmacochaperone (Jean-Alphonse et al., 2009). The MCF14 compound is a cell-permeable high-affinity nonpeptide agonist of the V2R. It was proven in previous studies that it can promote the maturation and rescue of certain V2R mutant variants (Jean-Alphonse et al., 2009). Moreover, it was demonstrated that MCF14 can directly activate the cAMP signaling of several V2R mutants. In addition, the MCF14-induced receptor activation did not initiate arrestin binding, receptor internalization, and consequent receptor downregulation, which can be therapeutically beneficial (Jean-Alphonse et al., 2009). Pretreatment of WT-V2R expressing HEK293 cells with 10 µM MCF14 caused an increase in the basal cAMP level (Figure 3B, green traces, before the agonist stimulation). This was barely inducible further by 1 µM AVP stimulation (Figure 3B, green filled circles) based on the measured BRET ratio changes using the cAMP sensitive Epac-BRET probe. Probably, the applied 10 µM concentration of MCF14 was difficult to eliminate even though extensive washing steps were performed, which was also reported by (Jean-Alphonse et al., 2009). Similar to tolvaptan administration, the S127F-V2R did not show any basal activity upon MCF14 pretreatment (Figure 3B, brown traces, before the agonist stimulation). The stimulation of MCF14 pretreated cells with 1 µM AVP resulted in a rapid and robust BRET ratio change reporting a significant intracellular cAMP signal generation (Figure 3B, brown filled circles). This indicates that the V2R agonist MCF14 is a pharmacochaperone, but not an activator of the S127F-V2R. The effective rescue action of MCF14 on S127F-V2R demonstrated that the compound was able to reach and bind to the intracellularly-trapped receptor molecules. Next, we investigated the possibility of whether the acute stimulation of S127F-V2R expressing HEK293 cells with MCF14 can activate directly the misfolded receptors (Figure 4). We demonstrated that 10 µM MCF14 was able to induce similar BRET ratio change as the 1 µM AVP stimulation in the WT-V2R cells (Figure 4, red and grey traces), but 10 µM MCF14 stimulation did not result in marked signal generation in the S127F-V2R cells (Figure 4, green trace).

### Determination of the Useful Concentrations of V2R Ligands to Initiate cAMP Generation in Response to Arginine-Vasopressin Stimulations

**FIGURE 5:**
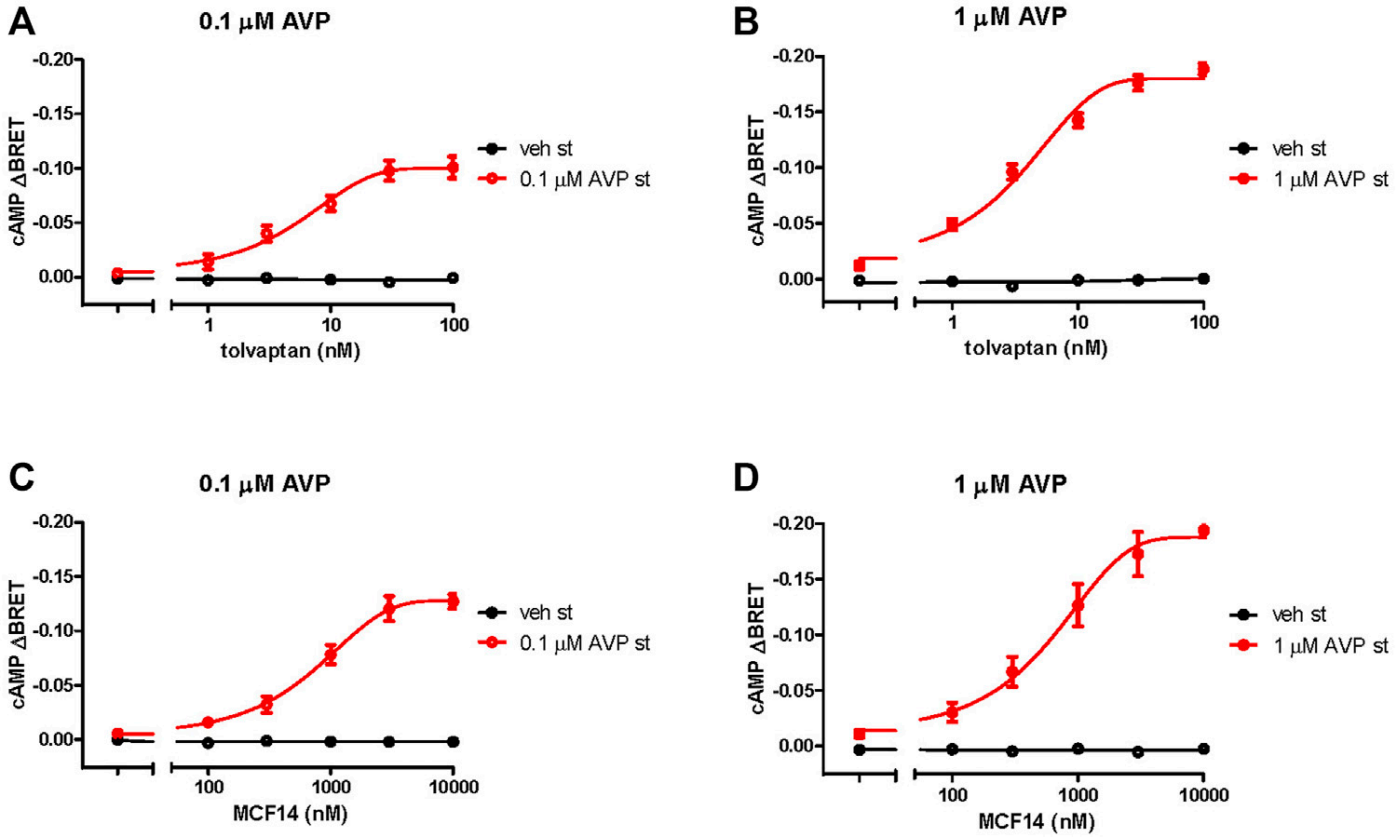
Effects of different concentrations of pharmacochaperone treatments on the cAMP generation of S127F-V2R. Effect of various **(A, B)** tolvaptan and **(C, D)** MCF14 pretreatment concentrations on the cAMP generation in response to 0.1 µM (**(A, C)**, red traces) or 1 µM AVP (**(B, D)**, red traces) stimulation. HEK293 cells were transiently transfected with the plasmids of the Epac-BRET sensor and S127F-V2R. 24 hours after the transfection the cells were incubated with different concentrations of tolvaptan or MCF14 for eighteen hours. The effect of stimulation was calculated as the BRET ratio difference (ΔBRET) between the BRET ratio values taken before and 5 min after the stimulation. The concentration-response sigmoidal curve was generated using non-linear regression with the GraphPad Prism software. Mean values ± S.E. are shown (*n* = 3). Since increasing cAMP decreases the BRET ratio, the y axis was inverted to visually represent cAMP level changes.

We examined whether the functional rescue of the mutant receptor might be achieved using lower concentrations of the pharmacochaperones (100 nM tolvaptan and 10 µM MCF14), compared to the levels, which were used in previous cell culture-based studies (Jean-Alphonse et al., 2009; Erdelyi et al., 2015). We determined the concentration-response curves of the functional rescue effect by tolvaptan and MCF14 on the S127F mutant receptor. According to our data, the previously used pharmacochaperone concentrations (100 nM tolvaptan and 10 µM MCF14) were sufficient to evoke the maximally obtainable effect on the cAMP generation capability of S127F-V2R and the used chaperone concentrations could be potentially decreased by half based on the concentration-response curve (Figure 5).

### Effects of V2R Pharmacochaperone Ligands on the Cell Surface Expression of the S127F-V2R

**FIGURE 6:**
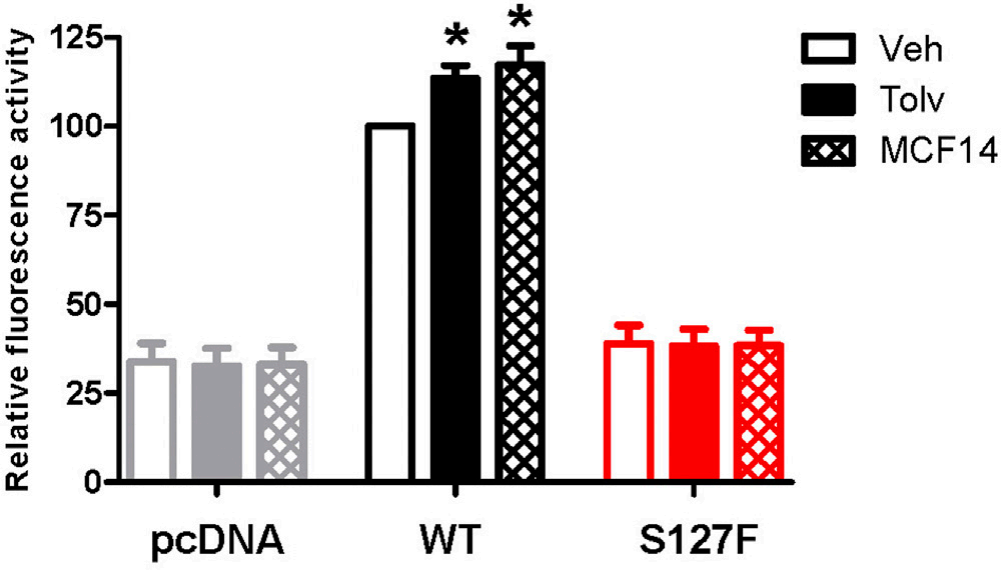
Measurement of the effect of pharmacochaperone treatment on cell surface expression of WT-V2R or S127F-V2R. HEK293 cells were transiently transfected with either HA-tagged V2-receptor construct or pcDNA3.1 and after 24 h of transfection, the transfected cells were treated with 100 nM tolvaptan or 10 µM MCF14 or vehicle (DMSO) for 18 h. Cell surface expression values of the immunofluorescent labeled receptors were analyzed and quantified by flow cytometry. Non-specific fluorescence was determined by measuring empty pcDNA3.1 transfected HEK293 cells incubated with anti-HA-Alexa 488 antibodies. The cell surface expression values are expressed as a percentage of the vehicle-treated V2R median fluorescence values done in the same experiment, each performed in triplicate. Mean values ±S.E. are shown (*n* = 3). Significance was calculated with one-way ANOVA test (*p < 0.05).

**FIGURE 7:**
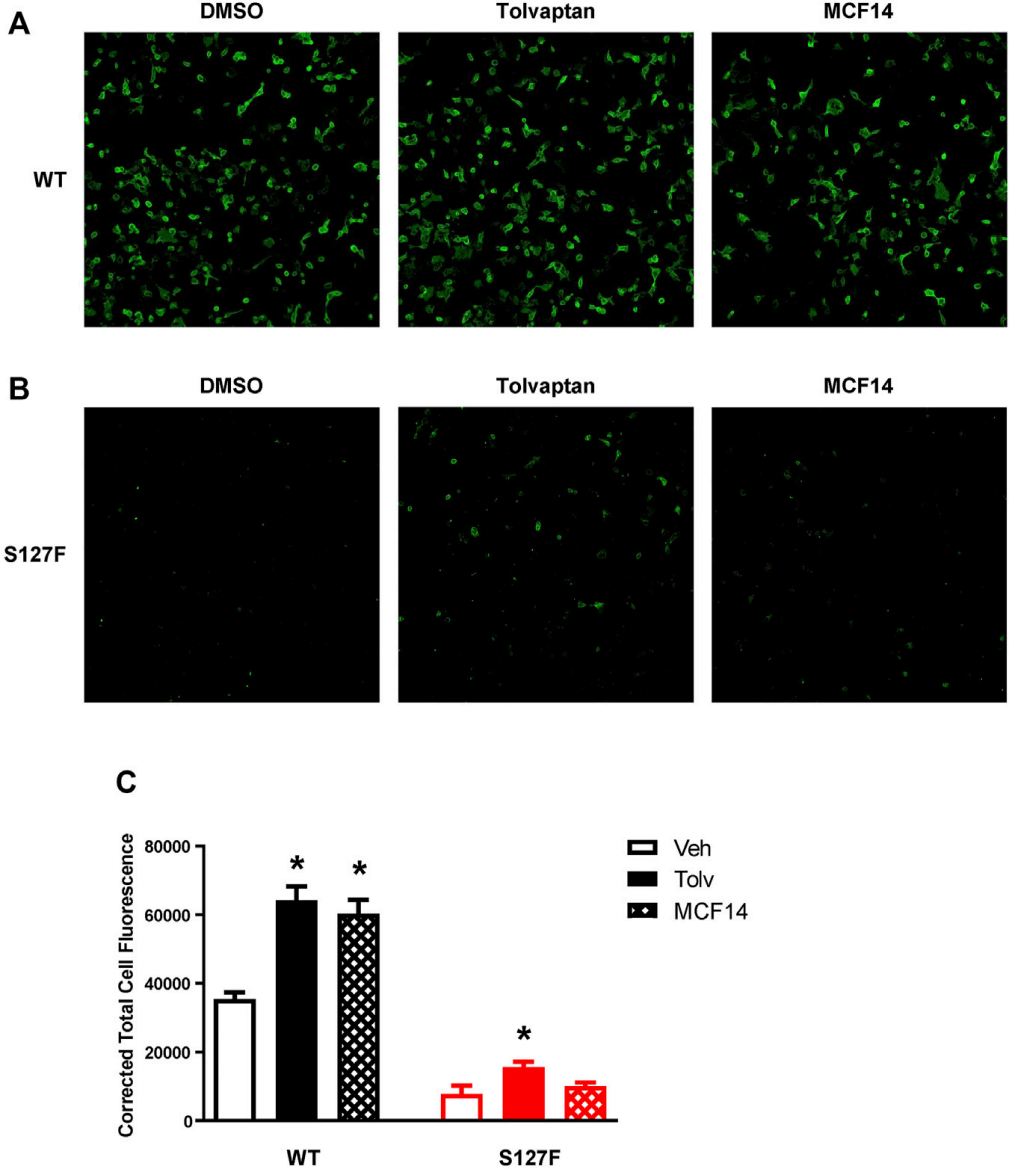
Visualization of cell surface expression of the WT-V2R or S127F-V2R after treatment with the pharmacochaperone V2R ligands. HEK293 cells were transfected with **(A)** WT-V2R-HA or **(B)** S127F-V2R-HA and 24 h after the transfection 100 nM tolvaptan or 10 µM MCF14 or vehicle (DMSO) was added to the medium of the cells for 18 hours. The HA-tag-containing receptor variants were directly immunostained with anti-HA-Alexa488 mouse monoclonal antibody on the cell surface (using non-permeabilized staining conditions). Imaging of cells was performed with ImageXpress Micro Confocal microscope system keeping the same exposure parameters (acquisition time and gain) between wells. The microscopic images were obtained by using MetaXpress software. **(C)** The cell fluorescence was determined by the CTCF method using ImageJ software. Significance was calculated with one-way ANOVA test (*p < 0.05).

The promising functional rescue of the S127F-V2R mutant by the application of V2R pharmacochaperone ligands raised the possibility that the rescued receptors were significantly translocated to the cell membrane. Therefore, we examined the cell surface expression of the receptors after 18 h of tolvaptan or MCF14 treatment. We measured the effect of tolvaptan or MCF14 treatment on cell surface expression using flow cytometry. HEK293 cells were transiently transfected with either WT-V2R or S127F-V2R and after 24 h of transfection, the cells were treated with 100 nM tolvaptan, or 10 μM MCF14, or vehicle (DMSO) for 18 h. According to our data, both tolvaptan and MCF14 treatments were able to slightly elevate the cell surface level of the WT-V2R (Figure 6). We could not detect any significant change in the cell surface expression level of S127-V2R, which may reflect that only a very small fraction of the intracellularly retained receptors were rescued by the tolvaptan or MCF14 treatment (Figure 6). We also investigated the effects of pharmacochaperone treatments in microscopy measurements staining the cell surface located receptors. We found that 100 nM tolvaptan and 10 µM MCF14 treatment induced significant and only weak accumulation of the cell surface level of the mutant receptors in microscopy determinations, respectively (Figure 7).

## Discussion

The congenital NDI is a rare disease and approximately 90% of the patients are males. The X-linked recessive mutations in the *AVPR2* gene result in defective V2R in the kidney epithelial cells (Bichet and Bockenhauer, 2016). It was reported in early works that the patients with congenital NDI do not respond to desmopressin treatment, suggesting that the cAMP signaling is defective in these cases (Bichet et al., 1989). The *AVPR2* gene is located on chromosome region Xq28. Shortly after the cloning of the gene, it was proposed that AVPR2 could be responsible for congenital NDI (Birnbaumer et al., 1992; Rosenthal et al., 1992; Seibold et al., 1993). In this study, we have identified and characterized an S127F (p.Ser127Phe) missense V2R mutation in a Hungarian family (Figure 1). This mutation was already identified in a Japanese family (Arthus et al., 2000). Since this disease-causing *AVPR2* mutation is present in at least two non-related families from distinct populations, we decided to characterize the cellular consequences and pathomechanism underlying this NDI-causing mutation. The DNA sequencing revealed that the Hungarian proband was hemizygous for the mutation, whereas his mother was heterozygous for the S127F mutation confirming her carrier status (Figure 1). The male proband shows the classical clinical symptoms of the NDI. As *AVPR2* mutations cause recessive X-linked NDI, thus male patients show severe clinical symptoms, while heterozygous females are less affected. The mild, subclinical symptoms of the heterozygous mother of the proband can be potentially further explained by the skewed X-chromosome inactivation. A few congenital NDI families were previously described in which the female family members exhibit variable, but usually mild symptoms of diabetes insipidus (Nomura et al., 1997; Van Lieburg et al., 1999; Carroll et al., 2006). These heterozygous female individuals possess both normal and mutated *AVPR2* alleles and it was suggested that the severity of symptoms was dependent on the rate of skewed methylation of the X chromosome. The NDI phenotype is caused by dominant methylation of the normal allele of the *AVPR2* gene (Nomura et al., 1997; Arthus et al., 2000).

We have characterized the properties of the loss-of-function S127F mutant receptor. In addition, we investigated the possibility to at least partially rescue the function of the S127F mutant V2 receptor. Based on microscopy analyses, we found that the expressed S127F-V2R is primarily located in intracellular compartments, probably due to ER retention caused by the misfolded mutant receptor structure (Figure 2D). Significant cell surface expression of the receptor was not detectable (Figures 2B, 7B). In line with the negligible presence in the plasma membrane, the overexpressed S127F-V2R mutant receptor has negligible signaling capability compared to the wild-type receptor upon vasopressin stimulation. Our highly sensitive, real-time BRET measurements revealed that the AVP stimulation of the S127F-V2R was able to generate a minuscule but statistically significant cAMP generation (Figure 2E).

Mutations in the GPCRs cause frequent misfolding and consequent intracellular retention of the receptors leading to diminished plasma membrane presence and reduced sensitivity to agonists. These cellular changes may lead to conformational or protein-misfolding disease (Cohen and Kelly, 2003), and the majority of the congenital NDI mutations fall into that category. Numerous missense, in-frame deletion, and insertion, last exon mutations are identified in the *AVPR2* gene which causes aberrant folding, and consequently, the misfolded mutant receptors are retained in the ER (Robben et al., 2006). Certain ER retention mutant misfolded proteins can be rescued by pharmacological chaperones (pharmacochaperones), and several V2R mutant variants were rescued by this approach previously (Morello et al., 2000; Tan et al., 2003; Bernier et al., 2004b; Wuller et al., 2004; Bernier et al., 2006; Jean-Alphonse et al., 2009). The concept of pharmacological chaperones can provide a new therapeutic approach to the functional restoration of impaired and ER retained receptors (Ulloa-Aguirre et al., 2004). With the help of pharmacological chaperones, the mutant receptors can be moved out of the ER to the cell surface. There, they might be capable of binding to their ligands and respond to hormone stimulation by initiating signal transduction (Romisch, 2004). Since the pharmacological chaperones are promising therapeutical candidates in the treatment of GPCR-associated misfolding diseases (Bernier et al., 2004a; Ulloa-Aguirre et al., 2004; Jean-Alphonse et al., 2009), we examined the potential rescue effects of such molecules on the intracellularly trapped S127F-V2R. Tolvaptan and MCF14 compounds can serve as pharmacochaperones for certain V2R mutants since they are not just specific nonpeptide V2R ligands but they can also cross the plasma membrane and potentially bind to the intracellularly retained V2Rs. Tolvaptan is a selective inverse agonist of V2R, which can block the constitutive and agonist-induced activation of the receptor. It is an approved medicine in the treatment of certain diseases such as hyponatremia, syndrome of inappropriate antidiuretic hormone (SIADH), and rapidly progressing autosomal dominant polycystic kidney disease (ADPKD) (Gattone et al., 2003; Goldsmith, 2005; Schrier et al., 2006; Starremans et al., 2008). Our observation using tolvaptan is in good agreement with other published data. Tolvaptan treatment was able to inhibit the basal (constitutive) activity of V2R (Figure 3A) and it can increase the cell surface expression of the transiently expressed wild-type V2R (Figures 6, 7) (Takahashi et al., 2012). Significant cell surface expression increment was detectable in S127F mutant receptor-expressing cells after tolvaptan pretreatment in microscopy (Figure 7) but not in flow cytometry experiments (Figure 6). Despite the lack of robust effect of tolvaptan pretreatment on receptor translocation from intracellular compartments to plasma membrane, Figure 3A demonstrates that tolvaptan pretreatment effectively rescued the cAMP generation capability of the S127F-V2R mutant in response to AVP (Figure 3A). The third transmembrane helix of the V2R (including the residue 127) is involved in the AVP binding (Slusarz et al., 2006a; Slusarz et al., 2006b), but the S127 is not in direct contact with AVP based on the 3D structure of AVP-V2R complex (Bous et al., 2021). Taken together, the impaired response to AVP stimulation is rather due to intracellular-endoplasmic reticulum retention but it cannot be excluded that both plasma membrane expression and AVP affinity may be modified by the S127F mutation. Tolvaptan pretreatment induced functional rescue of the S127F-V2R revealed that pharmacochaperones could be potentially used in the clinical practice in the treatment of S127F mutation-caused NDI cases. Although, the potential clinical use of tolvaptan in NDI could be limited since tolvaptan has been reported as a potential hepatotoxic compound (Torres et al., 2012). Moreover, its potential use would be limited by its antagonistic effect. To explore other possibilities, we have investigated the potential pharmacochaperone effect of the MCF14 compound, formerly known as Otsuka compound OPC23h, as it was shown earlier to rescue several V2R mutant receptors (Jean-Alphonse et al., 2009). MCF14 pretreatment alone was not able to induce any increase in the basal cAMP level, probably due to its inability to acutely activate the intracellularly trapped S127F mutant receptors (Figures 3, 4), but it was found as an effective pharmacochaperone for the S127F-V2R. The administration of 10 µM MCF14 for 18 h was able to functionally rescue the mutant receptor since the AVP stimulation extensively increased the intracellular cAMP level (Figure 3B). Surprisingly, in contrast to the functional rescue effects induced by tolvaptan or MCF14 pharmacochaperones, it was found that MCF14 treatment was not able to induce a significant increase in S127F-V2R cell surface expression (Figures 6, 7). This apparent discrepancy between the functional rescue and plasma membrane translocation results can be explained with the robust amplification in the signal transduction cascades (Brown et al., 1992; Buchwald, 2019). Signal amplification is an advantage of GPCR, including the G_s_ protein-coupled V2R induced signaling cascades (Keshelava et al., 2018). It is highly plausible that the pharmacochaperone treatment, either tolvaptan or MCF14 administration, caused the translocation of only a diminutive fraction of the intracellularly trapped receptors to the plasma membrane. This small degree of receptor translocation did not yield a significantly detectable increase in the cell surface receptor level in our experimental setups. Still, it was sufficient to functionally rescue the cAMP generation ability of the S127F-V2R expressing cells. A single V2R can activate multiple G_s_ proteins, each activating several downstream adenylyl cyclases that lead to the production of many cAMP molecules. Taken together, MCF14 seems to be a more potent starting compound for the development of a therapeutic agent in the treatment of S127F-V2R mutation-caused NDI. We did not detect differences between the signaling capability of tolvaptan and MCF14 rescued receptors, which shows that both the tolvaptan and MCF14 can bind the misfolded conformation of the S127F mutant receptor. However, MCF14 has advantages over tolvaptan since it is a functional-selective agonist without promoting hormone stimulation-induced arrestin binding and receptor-mediated endocytosis (Jean-Alphonse et al., 2009). The affinity of β-arrestin for the AVP-stimulated receptor determines the fate of the internalized receptor, such as the dephosphorylation and recycling back to the cell surface or the down-regulation of V2R by degradation. We tried to investigate the β-arrestin binding capability of the rescued S127F mutant receptor in response to vasopressin stimulation, but due to the very low level of plasma membrane S127F-V2R level (even after the pharmacochaperone treatments) the arrestin interaction was not detectable (data not shown).

In conclusion, our data show that S127F mutation in V2R leads to the production of intracellularly trapped receptors which can initiate cAMP signaling after their pharmacologically-induced translocation to the plasma membrane. Our results demonstrated that nonpeptide V2R ligands, such as tolvaptan and MCF14, can serve as pharmacological chaperone molecules to functionally rescue the S127F-V2R mutant receptor. These results suggest that pharmacological chaperones could be the ideal starting points in the development of therapy for NDI caused by class II mutations, including the S127F mutation of the V2 vasopressin receptor.

## Data Availability

The original contributions presented in the study are included in the article/Supplementary Materials, further inquiries can be directed to the corresponding authors.

## Abbreviations

AVP, arginine-vasopressin; AQP, aquaporin water channel; BRET, bioluminescence resonance energy transfer; cAMP, 3′,5′-cyclic adenosine monophosphate (cAMP), 1-deamino-8-d-arginine-vasopressin; ddAVP, desmopressin; GPCRs, G protein-coupled receptors; NDI, congenital nephrogenic diabetes insipidus; NSIAD, nephrogenic syndrome of inappropriate antidiuresis; Sluc, super *Renilla* luciferase; V2R, type 2 vasopressin receptor.
